# Supplementary material for: Heterozygous and generalist MxA super-restrictors overcome breadth-specificity tradeoffs in antiviral restriction
Source: bioRxiv. 2024 Oct 10:2024.10.10.617484. Preprint. [Version 1] doi: 10.1101/2024.10.10.617484 (PMC11482965; doi:10.1101/2024.10.10.617484)

Figure 1C

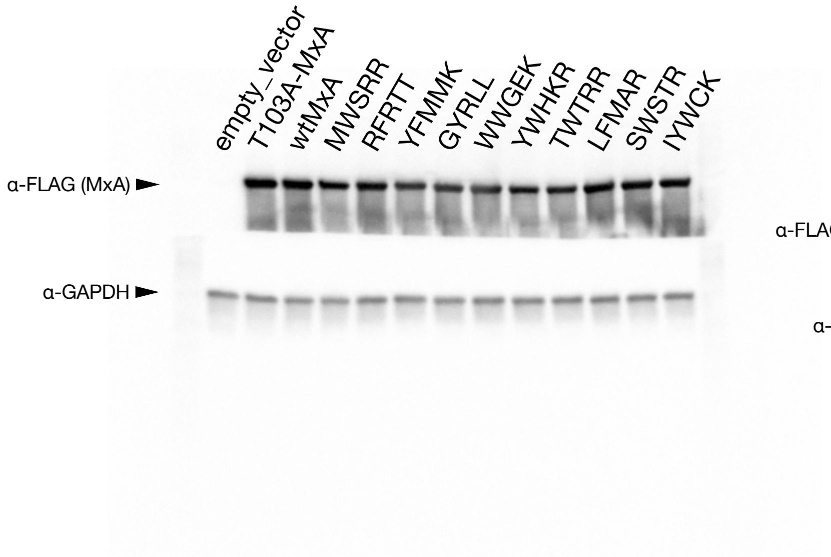

Figure 1D

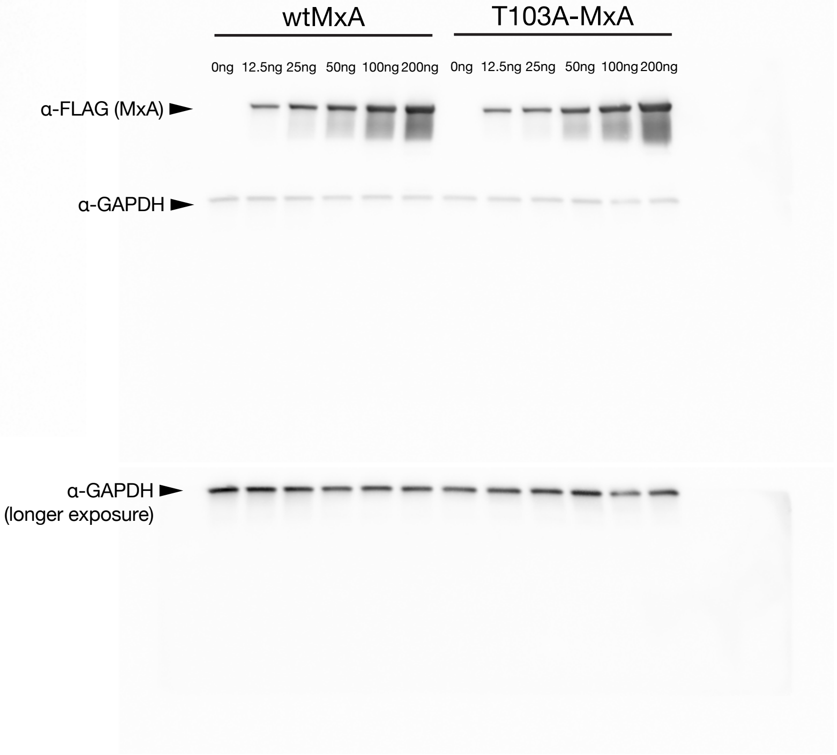

Figure 1D

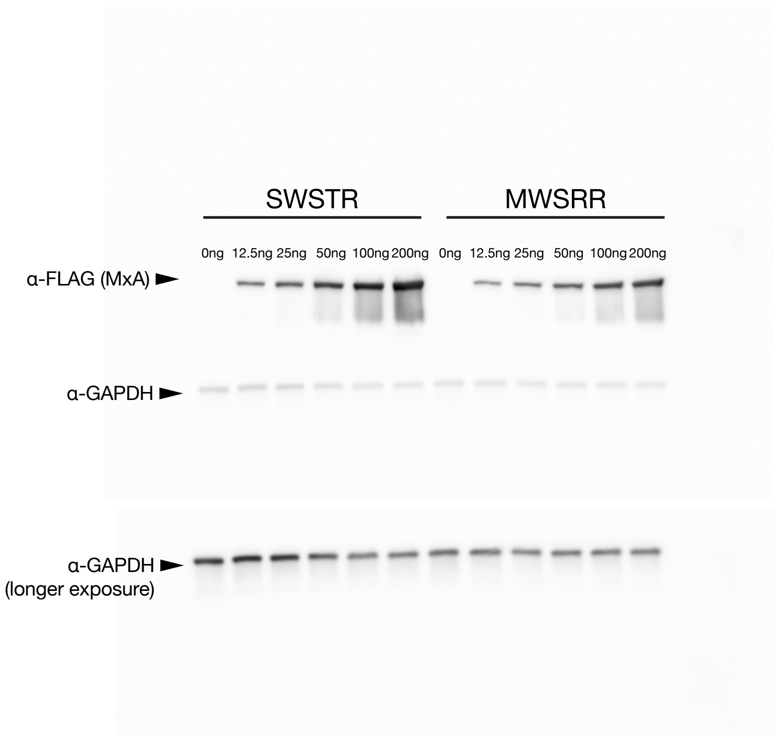

Figure 1D

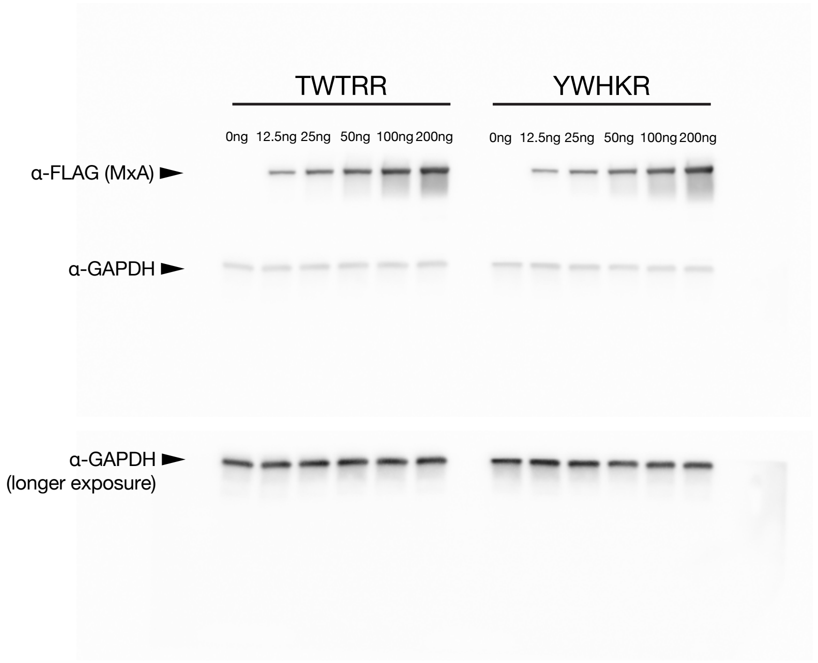

Figure S2B

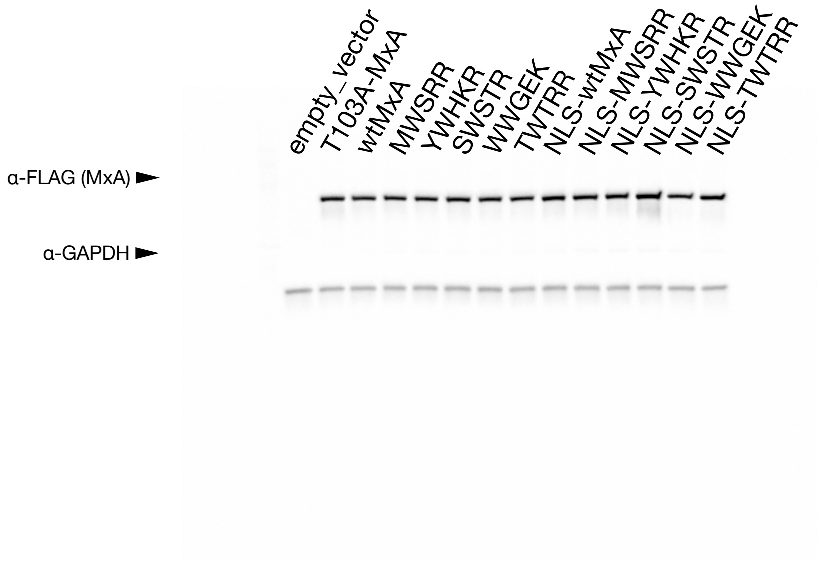

Figure 2C - left

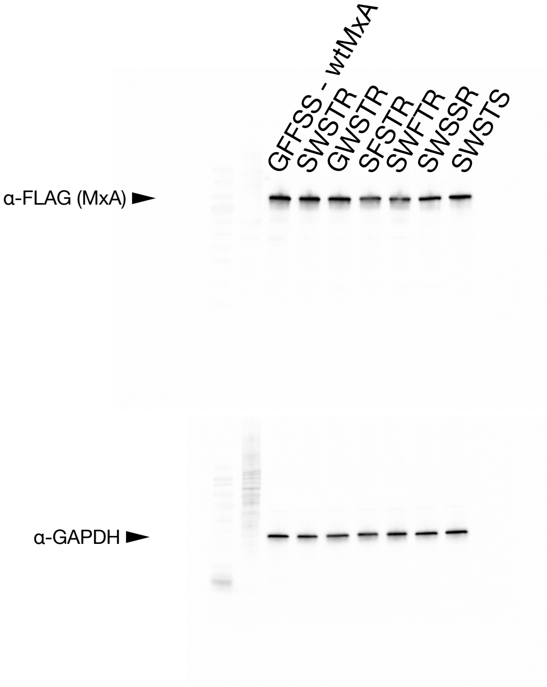

Figure 2C - right

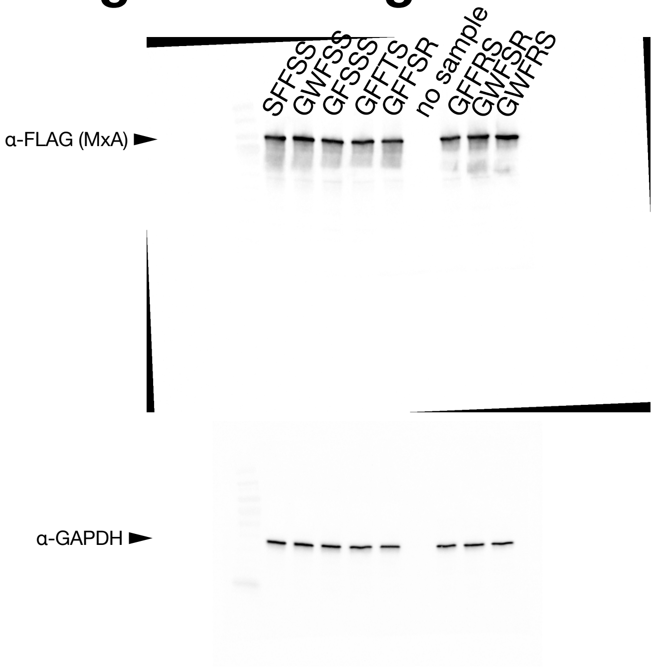

Figure 2D

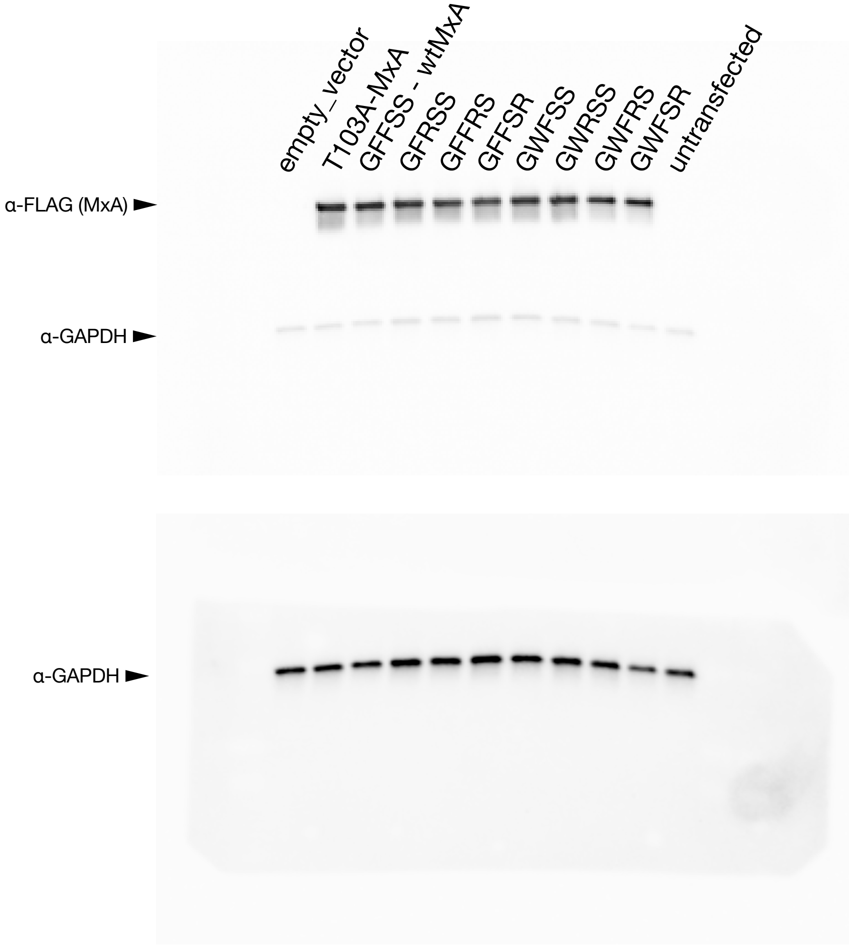

Figure S1

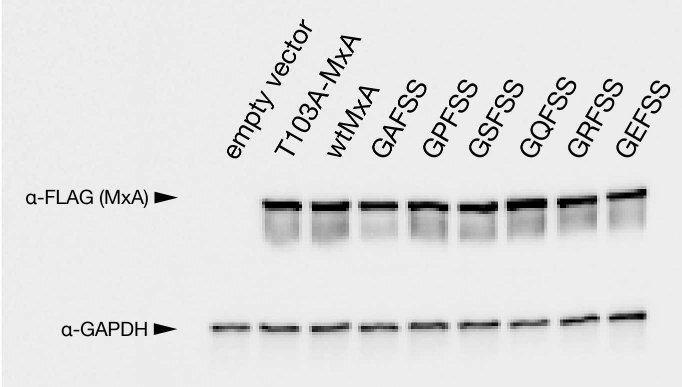

Figure 4A

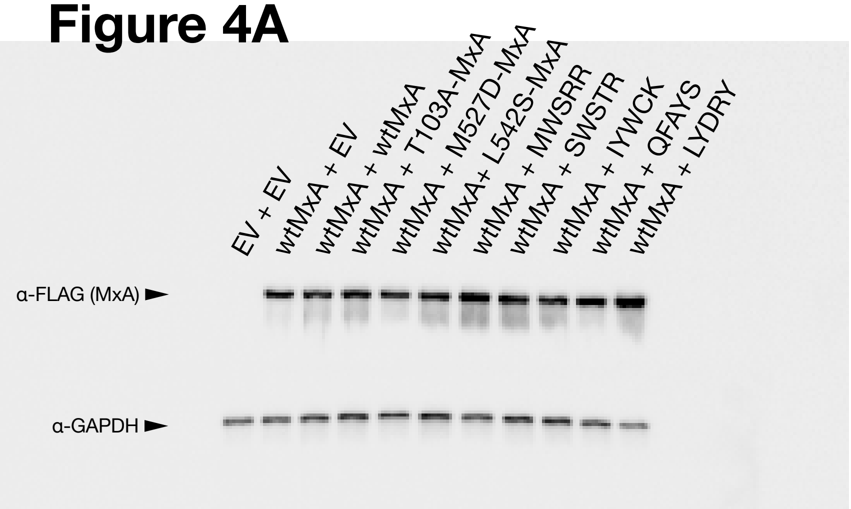

Figure 4C

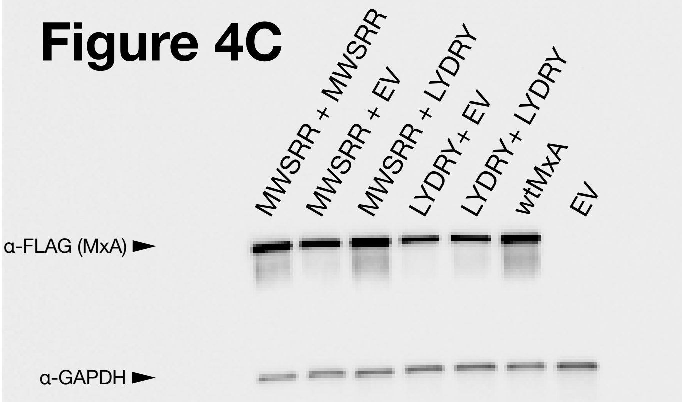

Figure S3A

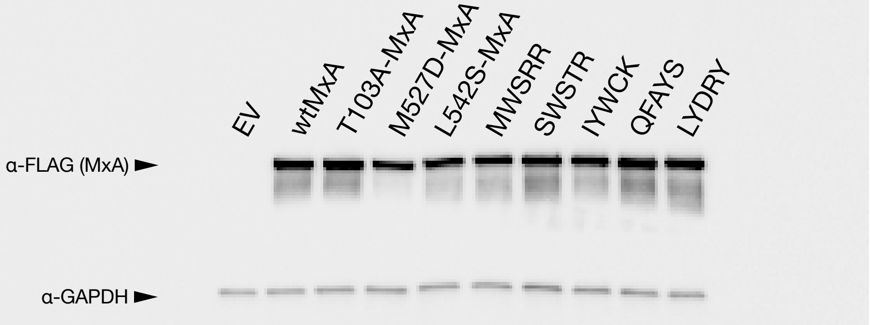

Figure 4B

Figure 4D

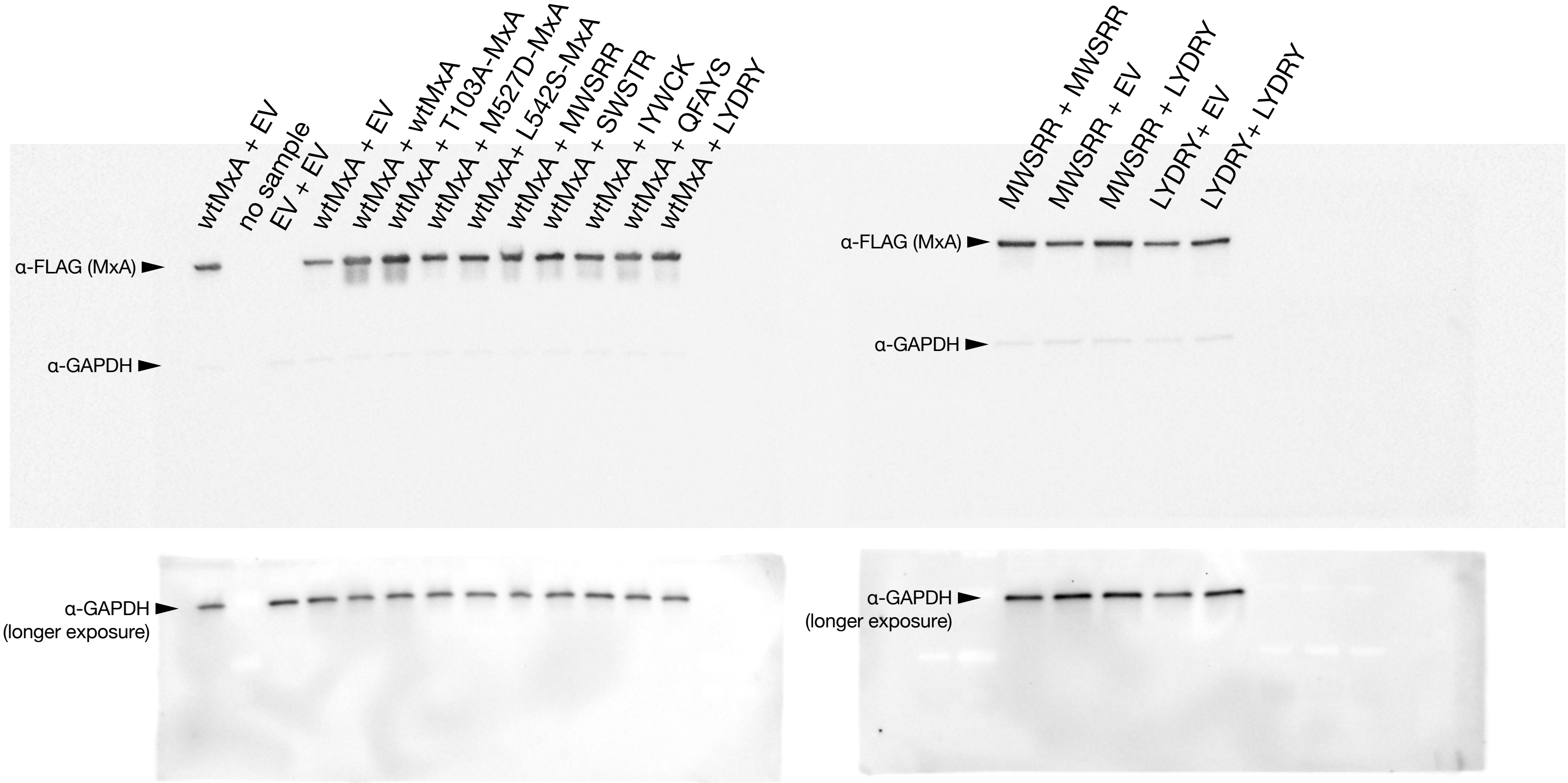

Figure S3B

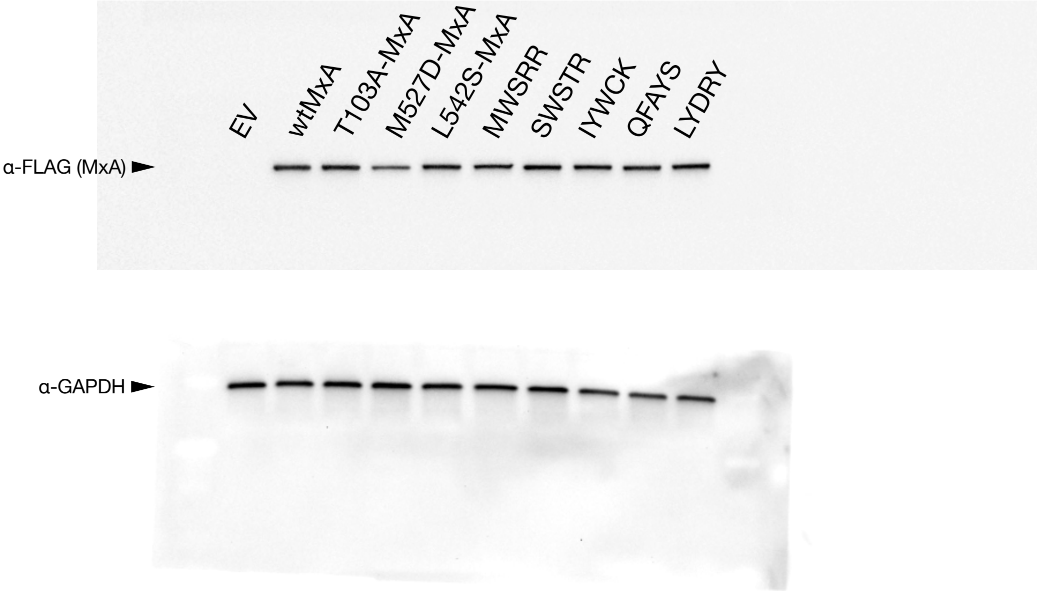

Supplement: Supplement 2 [file media-2.pdf]
