# Supplementary material for: Heterozygous and generalist MxA super-restrictors overcome breadth-specificity tradeoffs in antiviral restriction
Source: bioRxiv. 2024 Oct 10:2024.10.10.617484. Preprint. [Version 1] doi: 10.1101/2024.10.10.617484 (PMC11482965; doi:10.1101/2024.10.10.617484)
Supplement: Supplement 3 [file NIHPP2024.10.10.617484v1-supplement-3.pdf]

bioRxiv preprint doi: https://doi.org/10.1101/2024.10.10.617484; this version posted October 10, 2024. The copyright holder for this preprint (which was not certified by peer review) is the author/funder, who has granted bioRxiv a license to display the preprint in perpetuity. It is made available under aCC-BY 4.0 International license.

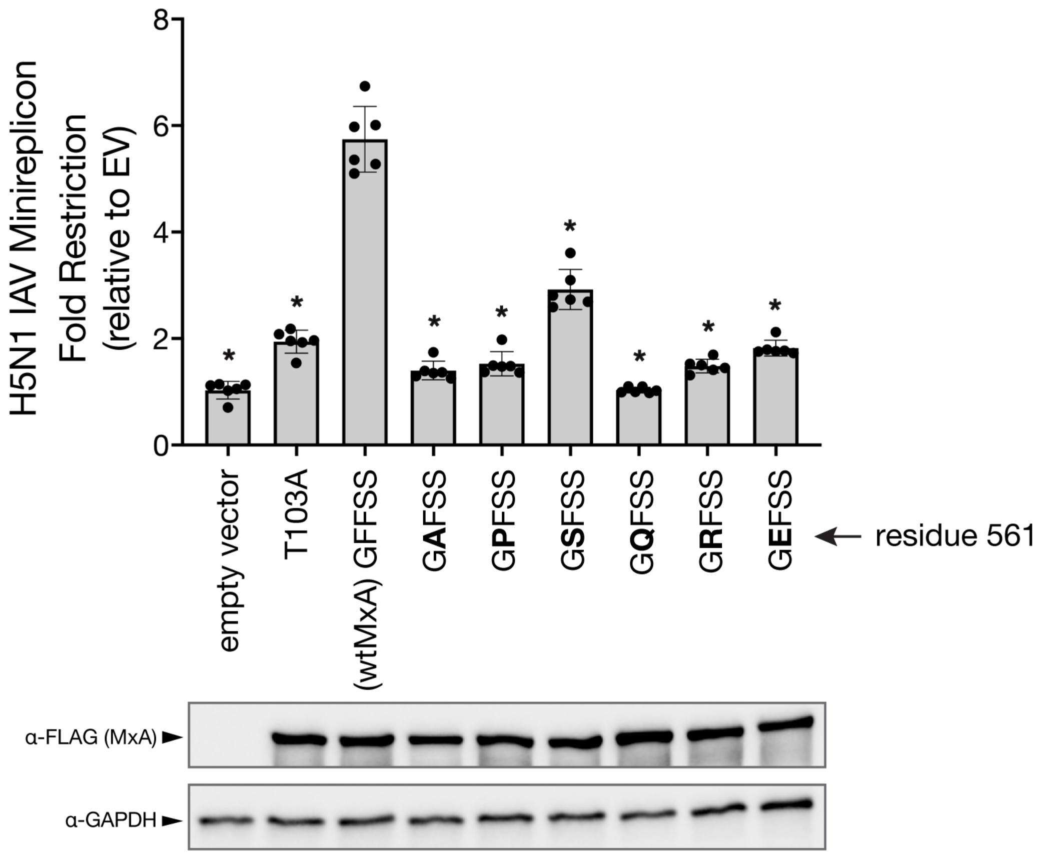

Figure S1: Restriction profiles of MxA variants with various non-aromatic amino acid residues at position 561. Fold restriction is reported relative to an empty vector. Each variant is labeled using amino acid identities at the five variable sites. We used unpaired Welch's t-tests between each variant and wtMxA to evaluate statistical significance (*p-value < 0.05).

bioRxiv preprint doi: https://doi.org/10.1101/2024.10.10.617484; this version posted October 10, 2024. The copyright holder for this preprint (which was not certified by peer review) is the author/funder, who has granted bioRxiv a license to display the preprint in perpetuity. It is made available under aCC-BY 4.0 International license.

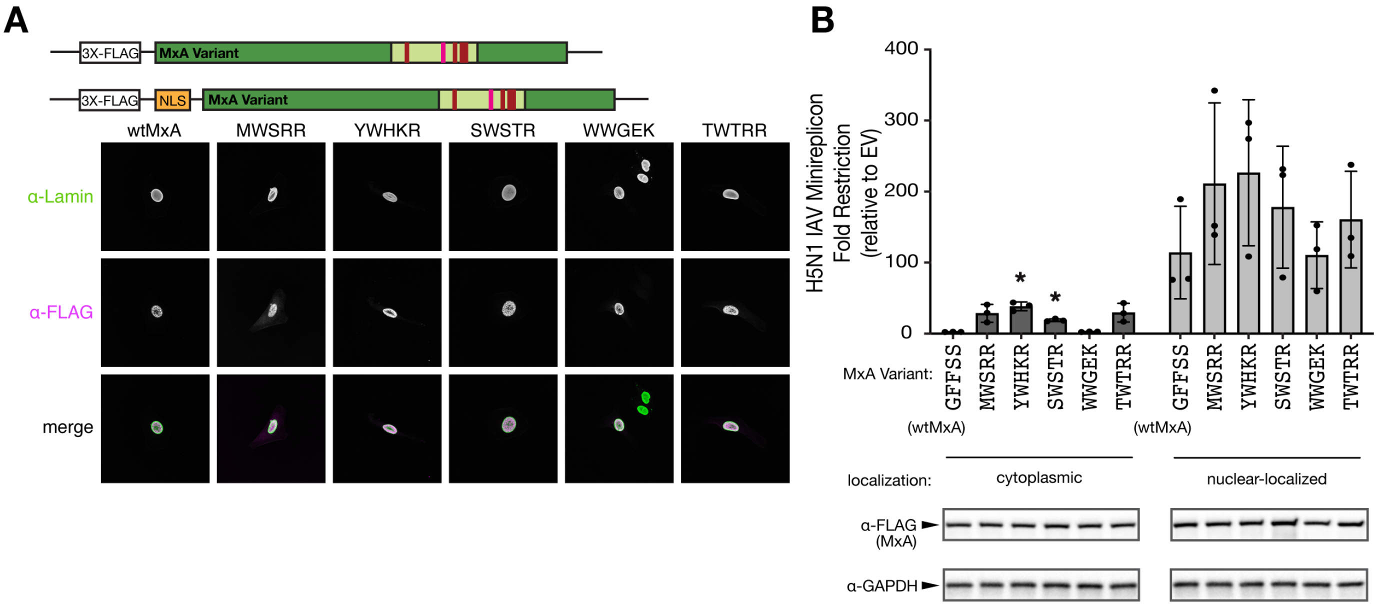

Figure S2: Nuclear localization further enhances H5N1 super-restriction. (A) The SV40 large T antigen nuclear localization signal (NLS) PKKKRKV was cloned into the N-termini of MxA variants between a 3X-FLAG tag and the MxA gene. NLS-tagged variants were transfected into HeLa cells and imaged in the same manner as described in Fig. 2A. **(B)** The five super-restrictor variants, as well as wtMxA, with and without an N-terminal NLS were assayed for their H5N1 restriction relative to an empty vector control in the minireplicon assay. Their expression levels were also tested by Western blotting. Unpaired Welch's t-tests were performed between restriction levels of cytoplasmic variants and wtMxA as well as between NLS-tagged variants and NLS-wtMxA (* $p < 0.05$).

bioRxiv preprint doi: https://doi.org/10.1101/2024.10.10.617484; this version posted October 10, 2024. The copyright holder for this preprint (which was not certified by peer review) is the author/funder, who has granted bioRxiv a license to display the preprint in perpetuity. It is made available under aCC-BY 4.0 International license.

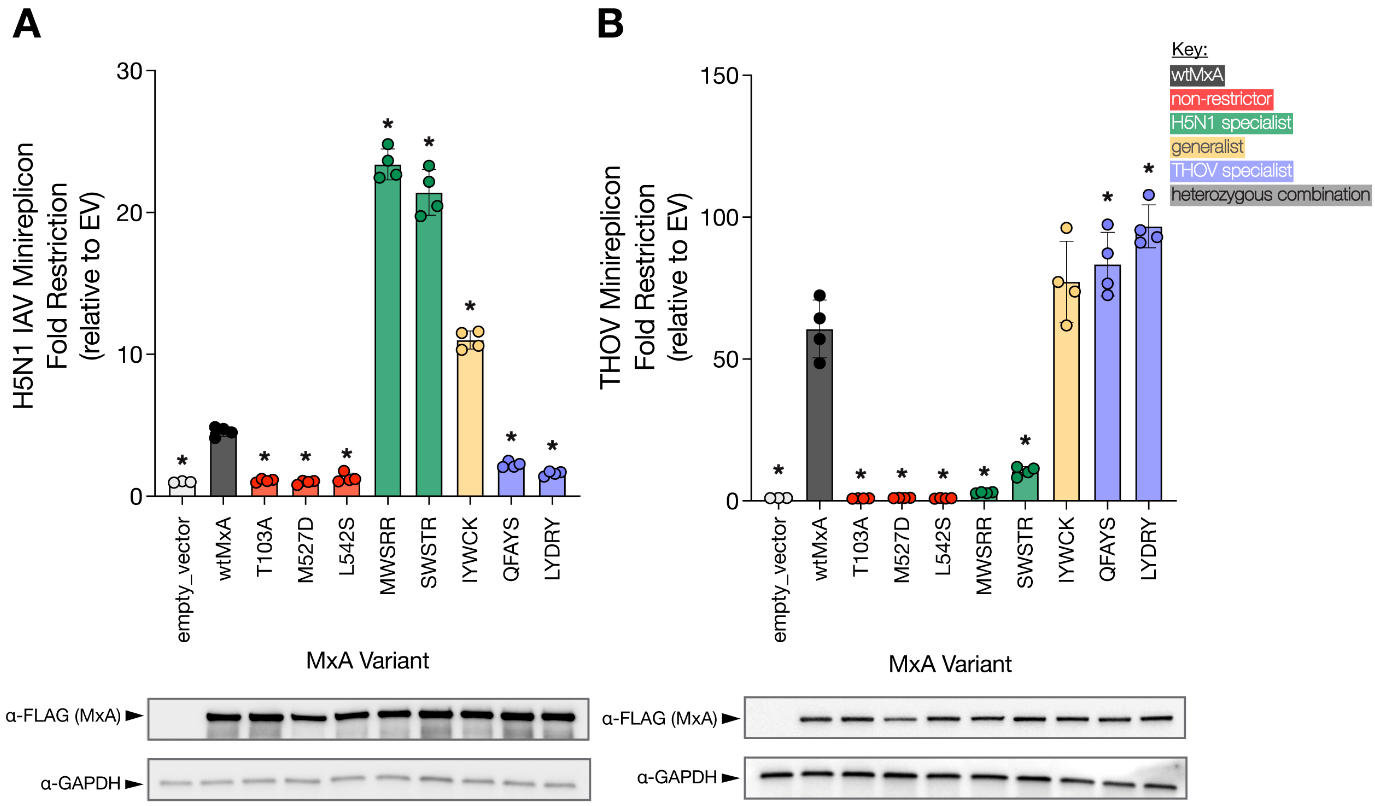

Figure S3: ‘Generalist’ and ‘specialist’ MxA variants restriction of H5N1 and THOV. We retested H5N1 **(A)** or THOV **(B)** restriction by wtMxA, non-restricting controls, specialist, and generalist MxA variants relative to an empty vector control based on a minireplicon assay; data is represented on a linear scale. The total amount of empty vector or MxA variant per condition is 100ng per well for H5N1 assay and 50 ng per well for the THOV assay. Unpaired Welch’s t-tests were performed between restriction levels of cytoplasmic variants and wtMxA as well as between NLS-tagged variants and NLS-wtMxA (* $p < 0.05$).
